# Community health workers at the dawn of a new era: 10. Programme performance and its assessment

**DOI:** 10.1186/s12961-021-00758-2

**Published:** 2021-10-12

**Authors:** Maryse Kok, Lauren Crigler, David Musoke, Madeleine Ballard, Steve Hodgins, Henry B. Perry

**Affiliations:** 1 Department of Global Health, KIT Royal Tropical Institute, Amsterdam, The Netherlands; 2 Crigler Consulting, LLC, Hillsborough, NC USA; 3 Department of Disease Control and Environmental Health, School of Public Health, Makerere University College of Health Sciences, Kampala, Uganda; 4 Community Health Impact Coalition, New York, NY USA; 5 Department of Global Health and Health Systems Design, Icahn School of Medicine at Mount Sinai, New York, NY USA; 6 School of Public Health, University of Alberta, Edmonton, AB Canada; 7 Department of International Health, Johns Hopkins Bloomberg School of Public Health, Baltimore, MD USA

**Keywords:** Community health workers, Performance, Monitoring, Primary healthcare

## Abstract

**Background:**

While the evidence supporting the effectiveness of community health worker (CHW) programmes is substantial, there is also considerable evidence that many of these programmes have notable weaknesses that need to be addressed in order for them to reach their full potential. Thus, considerations about CHW programme performance and its assessment must be taken into account as the importance of these programmes is becoming more widely appreciated. In this paper, the tenth in our 11-paper series, “Community health workers at the dawn of a new era”, we address CHW programme performance and how it is assessed from a systems perspective.

**Methods:**

The paper builds on the 2014 CHW Reference Guide, a compendium of case studies of 29 national CHW programmes, the 2018 WHO guideline on health policy and system support to optimize CHW programmes, and scientific studies on CHW programme performance published in the past 5 years.

**Results:**

The paper provides an overview of existing frameworks that are useful for assessing the performance of CHW programmes, with a specific focus on how individual CHW performance and community-level outcomes can be measured. The paper also reviews approaches that have been taken to assess CHW programme performance, from programme monitoring using the routine health information system to national assessments using quantitative and/or qualitative study designs and assessment checklists. The paper also discusses contextual factors that influence CHW programme performance, and reflects upon gaps and needs for the future with regard to assessment of CHW programme performance.

**Conclusion:**

Assessments of CHW programme performance can have various approaches and foci according to the programme and its context. Given the fact that CHW programmes are complex entities and part of health systems, their assessment ideally needs to be based on data derived from a mix of reliable sources. Assessments should be focused not only on effectiveness (what works) but also on contextual factors and enablers (how, for whom, under what circumstances). Investment in performance assessment is instrumental for continually innovating, upgrading, and improving CHW programmes at scale. Now is the time for new efforts in implementation research for strengthening CHW programming.

**Table:** 

Key messages: summary	
*Key findings*	
This paper addresses CHW programme performance and how it is assessed from a systems perspective • A variety of existing frameworks are useful when assessing the performance of CHW programmes • CHW programme performance assessments need to consider measurement of individual CHW performance and community-level outcomes, as well as look at health systems and other contextual factors that influence CHW programme performance	
*Key implications*	
• CHW programme assessments ideally need to be based on data derived from a mix of reliable sources and obtained using a mix of methods • Investment in CHW programme performance assessments is instrumental for continually innovating, upgrading, and improving CHW programmes at scale	

## Background

This is the tenth paper in our series, “Community health workers at the dawn of a new era”. Previous papers have provided an overview of national community health worker (CHW) programmes at present and the challenges they face (Paper 1 [1]), approaches to building partnerships, collaborations, and governance (Papers 2 [2] and 3 [3]), financing (Paper 4 [4]), roles and tasks (Paper 5 [5]), recruitment and training (Paper 6 [6]), supervision (Paper 7 [7]), motivation and reimbursement (Paper 8 [8]), and relationships with communities and with health systems (Paper 9 [9]). This series has been produced in recognition that CHW programmes are in a historical moment where the importance of their contribution to health systems and to population health improvement is becoming more widely recognized [10]. Rather than being an underfunded afterthought, CHWs need to be understood as an integral part—if not the foundation—of health systems, particularly (but not exclusively) in low- and middle-income countries (LMICs) [11, 12].

This historical moment has been produced by several powerful and concurrent circumstances. First, the evidence of the effectiveness of trained and supported CHWs in providing quality health services and improving population health continues to grow [13]. Countries that are making the strongest gains in improving the health of their populations have strong CHW programmes, such as Bangladesh, Brazil, Ethiopia, Nepal, and Rwanda [14]. Second, the Declaration of Alma-Ata [15], which was the first major global health document to assert the importance of CHWs in 1978, was reaffirmed on its 40th anniversary in the 2018 Declaration of Astana [16]. On this anniversary, the World Health Organization (WHO) for the first time created a guideline for optimizing the contribution of CHW programmes to health systems [10, 17]. The following year, the Director-General of WHO and the World Health Assembly strongly affirmed the importance of CHWs for achieving universal health coverage and strengthening primary healthcare (PHC) [18, 19]. Finally, the COVID-19 pandemic has clearly demonstrated the need for health workers at the community level who can prevent, detect, and respond [20, 21]. The pandemic has led to calls throughout the world for CHWs to receive appropriate respect and status (including pay), not to mention the personal protective equipment that is essential for them to carry out their duties safely [22]. The pandemic has also led to broad recognition of the importance of CHWs in high-income countries [23, 24].

Improved investment and integration of CHW programmes in the health sector in LMICs require evidence on performance. This again requires monitoring and evaluation. The performance of CHW programmes is influenced by support (or the lack thereof) from two sides—the health sector and the community. It is also influenced by the broader social, economic, and political context [25–27]. Performance can be measured at the individual CHW level as well as at the programme and population level [25, 27, 28]. As discussed in Paper 1 of this series, CHWs are embedded in the community health system, which is a subsystem of the overall health system [29]. This makes the assessment of overall CHW programme performance a complex undertaking because of the number of (formal and informal) actors involved and the many relationships and processes at play in improving health at the community level. It is therefore difficult to specifically attribute CHWs’ impact on health outcomes. Nevertheless, monitoring and evaluation of CHW programmes is of utmost importance to be able to continuously improve CHW programming and build more evidence on what works, why, and in which context [30].

**Table:** 

Key message 1	
While the assessment of CHW programme performance is a complex undertaking, it is of utmost importance to be able to continuously improve CHW programming	

## Methods

In this discussion paper, we address CHW programme performance and how it is assessed from a systems perspective, acknowledging that health systems are complex social arrangements [31]. We start by defining CHW and CHW programme performance, after which we reflect upon various frameworks that have been developed for considering the performance of CHW programmes. Next, we provide an overview of approaches to assessing CHW programme performance, and finally, we reflect upon identified gaps and needs for the future with regard to assessment of CHW programme performance.

This paper builds on previous work of our writing team, most notably the widely used 2014 CHW Reference Guide, which targeted CHW programme managers and policymakers, supporting them in developing and strengthening CHW programmes at scale [12]. It is also informed by a recently released compendium of case studies of 29 national CHW programmes [14]. In addition, the WHO guideline on health policy and system support to optimize CHW programmes (2018) is discussed in this paper, as this recent guidance not only aims to assist national governments and national and international partners in improving the design and implementation of CHW programmes, but also aims to improve their performance and evaluation [17]. We also discuss the CHW Assessment and Improvement Matrix (AIM) toolkit [32, 33]. Lastly, we include illustrative examples of studies on CHW programme performance and community health information systems (CHIS) published in the past 5 years (i.e., since publication of the CHW Reference Guide). For this purpose, we conducted a search on PubMed in August 2020, with a broad focus on CHWs, performance, information systems, and LMICs. We used a wide definition of CHWs, which includes a spectrum extending from volunteers with brief informal training to paid, professionalized CHWs, as outlined in Paper 1 of this series [1]. The 551 search results were screened on their titles and abstracts, and examples of partial or full CHW programme assessments, conducted with a variety of methods, were taken up in this paper to illustrate the discussion.

## Results

### Defining CHW and CHW programme performance

Well-performing health workers function in ways that are responsive, fair, and efficient to achieve the best health outcomes possible for clients, given the available resources and circumstances [34]. We use this definition of health worker performance for CHWs as well and stress that for CHWs to be responsive, fair, and efficient in achieving the best health outcomes in their communities, the available support from and relationships with actors in the health sector and the community are instrumental.

CHW performance at the individual CHW level is the sum of different interrelated attributes, such as knowledge, guideline adherence, competencies, motivation, job satisfaction, agency, attitudes, and self-esteem [25]. These attributes can be measured in various ways—some more difficult than others—and can thereby give an indication of CHW programme performance. However, CHW programme performance is not only the sum of performance-related individual attributes of CHWs; it is also defined by community-level outcomes, such as health service use and community empowerment, and, ultimately, health outcomes (impact) [25, 27, 28]. We will explore this in the next section.

### CHW programme performance frameworks

Over the past decade, several frameworks on CHW programme performance have been published. While these frameworks differ in terminology used and sometimes in focus, all address determinants of CHW individual and programme performance. We draw on three of these in this paper: Kok et al.’s framework of intervention design factors influencing CHW performance [25], Naimoli et al.’s CHW logic model [27], and Agarwal et al.’s conceptual framework for measuring CHW performance within PHC systems [28].

Agarwal et al. (2019) [28] recently developed a CHW performance measurement framework using the common input-process-output-outcome logic model approach, while recognizing that community health systems are nonlinear and complex. Determinants of CHW programme performance are presented as *inputs* and *programmatic processes*. The inputs include policies (e.g., CHW selection procedures and CHW roles and tasks), governance and stakeholders, logistics (e.g., transport and commodities), funding, and information management systems. Other widely used frameworks label these as health systems factors [25, 27]. The programmatic processes entail supportive systems (e.g., supervision, performance appraisal, and data use), CHW development (e.g. recruitment, training, and incentives), and support from community-based groups [28].

Naimoli et al. (2014) [27] label these as programme-level activities, where they distinguish technical support, social support, and incentives from the perspective of both health sector actors and community actors. In Kok et al.’s (2015) [25] conceptual framework on factors influencing CHW performance, these are labelled as intervention design factors; in Ballard and Montgomery’s (2016) systematic review, they are called intervention components [35]. These determinants of CHW programme performance are important, and we will come back to them later in this paper. We now focus on how performance can be defined and measured.

As indicated above, most frameworks make a distinction between individual CHW performance and community-level outcomes as indicators of CHW programme performance. Between the frameworks, the categorization of the attributes of individual CHW performance differs, but the attributes themselves are in line with each other. Following the categorization (indirect and direct) used in the work of Naimoli et al. (2014) [27], Table 1 contains the attributes of individual CHW performance from the three frameworks combined [25, 27, 28]. Some of these attributes slightly overlap. What is important, though, is how the attributes are operationalized, and this will vary by country and programme context. In the next section, we discuss how they could be measured.

**Table 1:** Indirect and direct attributes of individual CHW performance [25, 27, 28]

Category	CHW attribute	Definition
Indirect	Agency/empowerment	The CHW’s capacity to make purposeful choices and transform these into desired actions and outcomes (derived from the definition of empowerment by Petesch et al. [36])
	Adherence to guidelines	Degree to which the CHW adheres to programme guidelines
	Attitude	The CHW’s attitudes toward the client, community, other health workers, the employer, and organization for which s/he is working
	Competencies	Degree to which the CHW has the skills (e.g., in communication, diagnosis, treatment, referral, advocacy) necessary to carry out the tasks assigned to him/her
	Engagement	Degree to which the CHW enjoys and believes in what s/he does and feels valued for doing it [37]
	(Job) satisfaction	Degree to which the CHW derives personal satisfaction from his/her role/job
	Knowledge	Degree to which the CHW has the theoretical or practical understanding of the function and tasks assigned to him/her
	Morale	The mental and emotional condition (enthusiasm, confidence, etc.) of a CHW with regard to the function or tasks at hand
	Motivation	A CHW’s degree of willingness to exert and maintain effort on assigned tasks
	Self-esteem/efficacy	A CHW’s confidence, belief in his/her ability to produce an expected, desired result
Direct	Absenteeism	The rate at which CHWs do not carry out the assigned tasks
	Attrition	The rate at which practicing CHWs resign, retire, or abandon their positions
	Productivity	A CHW’s output of services/tasks
	Responsiveness	The degree to which a CHW responds to the needs of clients or groups within a reasonable time period
	Service delivery	Quantity of promotional, preventive, and curative services CHWs provide to community members
	Service quality	The degree to which CHWs provide promotional, preventive, and curative services according to quality standards and procedures

The three frameworks cover community-level outcomes as indicators of CHW programme performance. Naimoli et al. (2014) [27], unlike the others, distinguish CHW-attributable changes among individual clients, in the community, and in health systems. For example, CHWs could influence changes in health system structures, processes, and behaviours. In Table 2, we list community-level outcomes reported across the three frameworks. They include outcomes that can relate to individual community members and communities more broadly. Some of them overlap, and again, their usefulness depends on how they are operationalized in the context of a specific CHW programme.

**Table 2:** Community-level outcomes as measurements of CHW programme performance [25, 27, 28]

Outcome	Definition
Access	Equitable physical and social access to essential services delivered by CHWs, or delivery of services in a timely manner within the client’s home
Community functioning	(Changes in) a community’s structure, processes and behaviours resulting from its interaction with a CHW
Community empowerment	Degree to which both individuals and communities participate actively in community health activities
Cost savings	Money not spent by community members or by the community that otherwise would have been spent in the absence of a CHW
Credibility	Degree to which the community considers CHWs to be making an important and valuable contribution to the health and well-being of the community
Health-promoting behaviour	Community members or communities have adopted health-promoting behaviours at home or in the community as a result of contact with CHWs
Knowledge of service availability	Community members’ ability to identify the location of CHWs, the services they provide, and when they are available
Prestige	Status the community confers upon CHWs as a result of their selection and/or resulting from the quantity and quality of the services they deliver to community members
Referral/counter referral	Degree of acceptance and use of services provided at a health facility following referral by a CHW, and the degree of acceptance and use of CHW services when referred back to the CHW by the health facility
Satisfaction	Clients’ or communities’ reported degree of satisfaction with the services rendered by CHWs
Social cohesion	Change in the manner in which community members work towards achieving a goal or satisfy the emotional needs of its members (including marginalized or vulnerable groups) resulting from its interaction with a CHW
Trust	Degree to which community members or communities believe that the CHW will do his/her best to meet their needs [38]
Use of services/healthcare-seeking behaviour	Degree to which community members (including marginalized or vulnerable groups) with access to CHWs are routinely seeking and using promotional, preventive, and curative services that CHWs offer

**Fig. 1:**
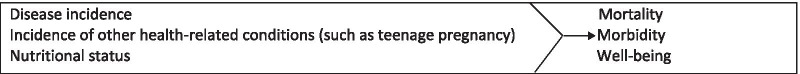
Impact at the population level of CHW programme performance

The performance of individual CHWs and the community-level outcomes will (ideally) lead to improved health outcomes at the population level (impact): equitable reductions in mortality and morbidity, improved well-being, reduced disease incidence, or changes in the incidence of health-related conditions (such as teenage pregnancy) (Fig. 1). While changes in population-level health indicators are readily measurable, attributing any observed changes to the impact of CHW programmes is a challenging methodological task and is often difficult in the absence of evaluations that have a control area where the CHW programme was not present. In addition, other influences that are producing measurable improvements in population health are also present, such as improved socioeconomic conditions and facility-based healthcare.

**Table:** 

Key message 2	
CHW programme performance assessments need to consider measurement of individual CHW performance, community-level outcomes, and impact at the population level	

### Approaches to assessing CHW programme performance

Depending on the programme and evaluation focus, a combination of the above-presented attributes of individual CHW performance and community-level outcomes can be included for assessing CHW programme performance. Different methods are suitable for this, depending upon the attribute or outcome and associated indicators.

#### The use of meeting minutes, training and accreditation logs, and reports from CHWs and supervisors

CHW programmes often have mechanisms for reporting which are different from data captured in the routine health information system. Minutes of meetings between CHWs and supervisors, or of village health or other committees involving CHWs, can provide insight into, for example, CHW satisfaction or productivity and their influencing factors. Training and accreditation logs, if available, can provide information about (gained) knowledge of CHWs. Supervisor reports and standardized checklists can include observations on guideline adherence and quality of care provided by CHWs [28, 39]. Supervisor reports can provide useful quantitative data if there is regular supervision of all CHWs, because infrequent supervision or supervision of only a part of the CHW workforce may distort indicator measurement [40].

There are various examples of studies in the recent academic literature in which these types of data are used to assess CHW performance, sometimes combined with data from other sources, such as surveys or qualitative interviews. These studies mostly focus on specific attributes of individual CHW performance in the context of vertical programmes. For example, in Nepal, Thapa et al. (2020) [41] assessed the knowledge of female community health volunteers (FCHVs) before and after a (cascade) training on postpartum family planning. They also reviewed FCHV registers, interviewed clients, and conducted focus group discussions with FCHVs. They concluded that the intervention resulted in increased knowledge and improved community-based counselling by FCHVs. However, long-term effects were not measured.

In Nigeria, Abimbola et al. (2016) [42] analysed the minutes of 129 community health committees (CHCs) across four states. These minutes provided a wealth of information about committee processes, deliberations, actions, decisions, and relations. CHCs had five modes of functioning in promoting community engagement in PHC. They provided forums (called village squares) for community stakeholders to interact with and support the health system, they reached out within their communities (community connectors), they lobbied governments for support (government *botherers*), they induced and augmented government support (back-up government), and took control of healthcare in their community (general overseers). While performing these functions, contextual factors, in particular power imbalances, limited their influence on improving health service delivery. It was therefore concluded that CHCs tended to promote collective action for self-support (of the committee) more than collective action for demanding accountability.

#### The use of information from the routine health information system

Key CHW programme activities and outputs need to be routinely documented and analysed, beginning at the most peripheral tier of the PHC system. The health information system provides a basis for this. However, the inclusion of community health services into the routine health information system varies by country. Depending on the available system, activities and outputs of CHWs can be specifically identified or not. For example, information systems may have data on referral from CHWs to health facilities. Other indicators, such as antenatal care (ANC) utilization, can be disaggregated based on whether it was offered at a community or facility level. Calculations to measure performance can then be made by comparing ANC use to the use based on the number of expected pregnancies in the CHW catchment area. If disaggregation between facility- and community-level data is not possible, it is more difficult to assess CHW programme performance.

The recent academic literature provides examples of studies that used data from routine health information systems to assess CHW performance. In Mozambique, Jose et al. (2020) [43] evaluated the role of CHWs in promoting facility-based tuberculosis (TB) screening and household contact tracing using routine national TB programme data in five intervention and seven control districts. They concluded that the involvement of CHWs led to an increase in TB notifications over the period of 1 year. In Sierra Leone, CHW reports were discussed in community health data review meetings where community leaders, members of ward development and health management committees, CHW peer supervisors, and representatives of the peripheral health unit assessed CHW performance and developed action plans for improvement [44].

Roberton et al. (2016) [40] examined the feasibility of collecting integrated community case management (iCCM) routine indicators as included in the iCCM Indicator Guide in 10 countries. They concluded that the majority of data needed are being collected through existing monitoring systems. However, they are mostly available at the health facility level and do not get aggregated at district or national level [40].

#### The use of information from existing periodic national surveys

National demographic and health surveys (DHSs) include indicators that provide valuable information on CHW programme performance. For example, DHS 8 woman’s and man’s questionnaires contain indicators such as the percentage of women visited by a CHW in the last 12 months and the percentage of men who discussed family planning with a CHW [45]. The United Nations Children’s Fund (UNICEF) multiple indicator cluster surveys also contain questions on CHWs. For example, the questionnaire for individual women covers whether CHWs offered antenatal, delivery, and postnatal care [46]. These data can be assessed over longer periods of times (e.g., every 5 years) to get a sense of how CHW programme performance is evolving.

#### Periodic internal or external CHW programme assessments

Countries can readily carry out their own periodic assessments of their national CHW programmes. One possible approach to this is national evaluation platforms (NEPs) that have been developed in some countries. NEPs build national capacity to systematically identify, compile, and rigorously analyse data from diverse sources to evaluate the effectiveness of maternal, newborn, and child health and nutrition programmes [47]. Efforts such as these could be adapted to assess national CHW programme performance. There are examples of countries that, often with funding from external donors, contract firms to carry out independent assessments of national CHW programmes as is commonly done with other development projects. For example, the health extension programme in Ethiopia was evaluated in 2018–2019 [48]. The national assessment consisted of a comprehensive mixed-methods study, a systematic review, and a participatory process to synthesize evidence and develop recommendations. The assessment revealed the strengths and weaknesses of the programme. Amongst others, it found gaps in health extension worker competencies as a result of suboptimal pre-service training. In 2011, a mixed-methods national assessment of CHW services in Afghanistan was conducted. It was found that training, supervision, and supplies were adequately organized, mainly by implementing nongovernmental organizations, but overload and motivation of CHW were issues of concern [49]. Other examples of national CHW programme assessments are provided in Table 4 (Appendix).

#### The use of primary studies and research tools

Primary studies often focus on a limited set of attributes or outcomes related to CHW programme performance. In addition, they often look at vertical CHW programmes. They are seldom applied to national and scaled CHW programmes. As mentioned in Paper 1 of this series, evidence from small-scale studies can still be relevant for large-scale programmes.

Primary studies can have various methodologies. Randomized controlled trials (RCTs) often assess the effect of a change in supportive systems, such as certain modalities of CHW supervision or incentives, on attributes of individual CHW performance, such as guideline adherence or productivity [50, 51], or community-level outcomes such as service use [52]. While RCTs are needed for robust evidence, the RCT design is relatively unhelpful in providing full assessments of complex programmes at scale. Comprehensive programme case studies are a useful addition, as well as implementation research, using surveys over time, scales, qualitative methods, and systems-thinking tools [17].

When using surveys to measure attributes or outcomes on CHW (programme) performance, researchers can consider numerous indicators, according to the CHW programme under study and its context. Agarwal et al. (2019) [28] provide example indicators in their work based on a review of peer-reviewed articles, reports and global data collection tools, and consultations with global stakeholders, community health implementers, advocates, measurement experts, and ministry of health representatives across seven countries. One possible indicator of productivity and the use of services is the household visitation rate. Mwanza et al. (2017) [53] reported on CHW supervisors using the lot quality assurance sampling (LQAS) methodology to evaluate CHW performance in terms of household visitation rate in Zambia. LQAS proved to be a useful tool to assess CHW performance and could be used for performance appraisal as well. Smittenaar et al. (2020) conducted large-scale surveys including recently delivered women, their husbands, mothers-in-law, and accredited social health activists (ASHAs) in Uttar Pradesh, India. Household visitation timing and rate, ASHAs’ knowledge and beliefs, and the content and target of their advice were found to influence maternal and newborn health-promoting and health-seeking behaviour [54].

With regard to scales on attributes of individual CHW performance, one can adjust existing scales developed for other purposes unrelated to CHWs to the context of CHW programmes. The engagement survey for measuring the well-being of workers is one example [55]. The engagement survey asks employees to rate items on: their belief in their work and organization; their belief in their ability to succeed; their relations with colleagues and their supervisor; their opportunities for professional advancement; the support and recognition they receive; and the influence they have in decision-making. Another valuable toolkit that can be used to measure attributes of individual CHW performance is CUBES, which focuses on contextual and perceptual drivers (including beliefs, such as self-efficacy) of behaviour [56].

Recently, a validated scale for measuring motivation specifically for CHWs was published, consisting of 12 items related to job satisfaction, organizational commitment, community commitment, and work conscientiousness [57]. Qualitative methods, along with quantitative scales or as a stand-alone method, can provide insight into CHW performance as well.

For example, Kane et al. (2016) [58] presented findings from six country case studies from Africa and Asia regarding CHW empowerment. Their analysis was based on Lee and Koh’s analytical framework of empowerment that identifies four dimensions of empowerment: meaningfulness, competence, self-determination, and impact. The study found CHWs had feelings of empowerment as well as disempowerment. The authors recommend that more attention should be given to the experience of CHWs when aiming for improving CHW programme performance. We fully support this statement, as it is obvious that addressing issues of concern to CHWs themselves is an essential strategy for improving overall CHW programme performance. Vareilles et al. (2015) [59] conducted a realist evaluation on motivation of community health volunteers of the Red Cross in Uganda. They describe how a supportive environment positively influenced three key drivers of volunteer motivation: feelings of autonomy, competence, and connectedness.

For measurement of community-level outcomes, community scorecards can be used, where communities score on performance indicators [60]. With this method, outcomes such as satisfaction with CHW services, and communities’ trust in and perceptions of credibility of CHWs can be assessed. In addition, a variety of qualitative methodologies can be used.

**Table:** 

Key message 3	
Approaches to assessing CHW programme performance can include monitoring based on available data in the health system, and primary studies or national programme assessments that use a variety of methods	

### CHW programme assessments: a focus on programmatic determinants of performance

Programme inputs, as mentioned before, entail policies, governance and stakeholders, logistics, funding, and information management systems. Programmatic processes entail supportive systems, CHW development, and support from community-based groups. Many studies focus on these inputs and processes—either one or all of them—because they influence CHW programme performance. Therefore, regular assessment of these programmatic determinants of performance provides information on whether a CHW programme is designed and being implemented in such a way as to yield good performance.

#### The WHO guideline

Based on available global evidence—including a systematic review of published literature reviews [61], 15 systematic reviews of relevant primary studies, and a stakeholder perception survey—the 2018 WHO guideline on health policy and system support to optimize CHW programmes [10, 17] provides recommendations on 15 programmatic determinants of performance. These are: selection of CHWs, duration of their pre-service training, competency domains for the curriculum for pre-service training, modalities of pre-service training, competency-based certification, supportive supervision, remuneration, contracting agreements, career ladder, target population size, data collection and use, types of CHWs, community engagement, and mobilization of community resources. Depending on the certainty of evidence about positive effects on CHW programme performance, WHO provides conditional or strong recommendations about the design of these programme components. While most recommendations are conditional, a strong recommendation is made for establishing robust community engagement strategies. WHO also strongly recommends remunerating practising CHWs for their work with a financial package commensurate with the job demands, complexity, number of hours, training, and roles that they undertake; and providing paid CHWs with a contracting agreement specifying role and responsibilities, working conditions, remuneration, and workers’ rights. Although evidence regarding the benefits of remuneration was of low certainly, these recommendations were made based on human and labour rights [17].

#### The CHW AIM toolkit

While the WHO guideline provides recommendations and considerations for optimization of the design, implementation, and performance of CHW programmes, it does not provide specific tools or checklists to assess the extent to which a current programme design can yield optimal performance. The often used CHW Assessment and Improvement Matrix (AIM) toolkit, developed in 2011 and updated in 2018, serves this goal [32, 33]. The updated toolkit contains a programme functionality matrix with 10 programme components that influence performance of CHW programmes: role and recruitment, training, accreditation, equipment and supplies, supervision, incentives, community involvement, opportunity for advancement, data, and linkages to the national health systems (Table 3). It is clear that these 10 programme components cover the 15 components included in the WHO guideline, as listed in the previous paragraph.

**Table 3:** Programmatic components of the updated CHW AIM tool [63]

	Component	Description
1	Role and recruitment	How the community, CHW, and health system design and achieve clarity on the CHW role and from where the CHW is identified and selected
2	Training	How pre-service training is provided to the CHW to prepare for his/her role and ensure s/he has the necessary skills to provide safe and quality care; and how ongoing training is provided to reinforce initial training, teach CHWs new skills, and to help ensure quality
3	Accreditation	How health knowledge and competencies are assessed and certified prior to practicing and recertified at regular intervals while practicing
4	Equipment and supplies	How the requisite equipment and supplies are made available when needed to deliver expected services
5	Supervision	How supportive supervision is carried out such that regular skill development, problem solving, performance review, and data auditing are provided
6	Incentives	How a balanced incentive package reflecting job expectations, including financial compensation in the form of a salary, and nonfinancial incentives, is provided
7	Community involvement	How a community supports the creation and maintenance of the CHW programme
8	Opportunity for advancement	How CHWs are provided career pathways
9	Data	How community-level data flow to the health system and back to the community and how they are used for quality improvement
10	Linkages to the national health system	The extent to which the ministry of health has policies in place that integrate and include CHWs in health system planning and budgeting and provides logistical support to sustain district, regional, and/or national CHW programmes

Each of the 10 components is subdivided into four levels of functionality: 1 = nonfunctional, 2 = partially functional, 3 = functional, and 4 = highly functional. For each of the programme components, each functionality level is described by situations commonly seen in CHW programmes, so that assessors can match the status of the CHW programme components against these descriptions to guide their assessment. For example, with regard to the component supervision, it goes from CHWs not or very occasionally being visited by supervisors or other health staff (score 1) to monthly, supportive supervision by dedicated and trained supervisors (score 4). The CHW AIM toolkit has been used by governmental, nongovernmental, and other stakeholders in the assessment, improvement, and planning of CHW programmes. Recently, it was used for the evaluation of the CORE Group Polio Project over a 20-year period in five countries. The successful programme, which made use of community mobilizers (CHWs), met the basic level of functioning (level 3) for six of the 10 components of the AIM tool [62].

#### Other ways to assess programmatic determinants of performance

Assessment of logistics and human resources can be informed by data on commodity availability from the logistics information system and by data on, for example, training and certification of CHWs from the human resources information system. Recently, a validated scale on perceived CHW supervision was published, containing six items that cover perceptions of CHWs on how they are supervised [64].

**Table:** 

Key message 4	
The 2018 WHO guideline [17] (2018) and the AIM toolkit [32, 33] provide guidance to assess a list of main programme components that influence CHW programme performance	

### Contextual factors influencing CHW programme performance

As stated in the Background of this paper, CHW programme performance is also influenced by the broader context. While it is not possible to change the context, at least in the short term, it is important to understand how certain contextual factors influence CHW programme components, attributes of individual CHW performance, and community-level outcomes. This can contribute to adjusting the programme to better fit in or respond to the context, resulting in better programme performance.

Kok et al. (2015) [65] conducted a literature review on contextual factors that influence CHW performance. The factors were categorized into the community context, economic context, environment, health system policy, and health system practice. The community context includes sociocultural factors (such as gender norms and values as well as disease-related stigma), safety and security, and knowledge of the target group. The environment includes geography and climate. The health system policy includes the existence of a CHW policy, human resource policy, legislation related to CHWs, and political commitment. Finally, a set of health system factors plays a role in influencing performance (health service functionality, human resource provisions, level of decision-making, costs of health services, and the governance and coordination structure). These factors should be taken into account when assessing CHW programme performance. As a recent example, Marston et al. (2020) [66] used qualitative research to provide insights into how family planning services offered by FCHVs in Nepal could be more tailored to the community context. For example, the study showed the importance of FCHVs upholding secrecy and privacy in their interactions with women to increase health-seeking behaviour. Bergström et al. [67] developed the Context Assessment for Community Health (COACH) tool, which is based on the PARIHS [Promoting Action on Research Implementation in Health Services] framework [68]. The COACH tool can be used to map and measure aspects of the local healthcare context that can influence the implementation of evidence-based interventions, including CHW programmes, in LMICs [67].

**Table:** 

Key message 5	
It is important to understand how contextual factors influence CHW programme performance, so that programme adjustments, aiming at tailoring the programme to the context, can be made to optimize performance. Contextual factors of importance are best identified through qualitative research	

### Gaps and needs for the future

This paper has focused on CHW programme performance and how this could be assessed. Performance assessments will differ by programme, as programmes have different features, in particular with regard to the types of CHWs, the services they provide, and their support mechanisms. As such, standardization of assessments of CHW programme performance is only possible to a certain extent. Given the fact that large-scale CHW programmes are complex entities and part of health systems, their assessment ideally needs to be based on data derived from a mix of reliable sources.

While it is useful and efficient to use existing data sources, such as routine health information systems, these sources have limitations. We already pointed out that in many settings, community-based data cannot be disaggregated from facility-based data. Routine health information systems often fail to capture data on services provided solely at a community level [30], and human resource information systems similarly do not always capture data on CHWs, especially when they are volunteers.

In addition, there are indications that data are not always reliable. Data collectors, including CHWs, sometimes see data collection and reporting as a “tick-the-box exercise”, because it concerns mere reporting up into the administrative *ether* rather than reporting data that are used at different levels to improve services delivery [69]. Feedback loops are often lacking. Regeru et al. (2020) [70] found that in Kenya and Malawi, there were large discrepancies between data reported by CHWs and those reported by their supervisors. Barriers to the reporting of high-quality data were (1) unavailability of standard data collection and reporting tools, (2) limited training of CHWs on data reporting, (3) parallel reporting requirements of vertical programmes, and (4) overload of CHWs tasks leading even to fabrication of data. In the study in Sierra Leone mentioned earlier, it was also found that CHW reported morbidity and mortality data were of low quality and incomplete [44].

CHISs need strengthening. Data quality assessments can stimulate improvements, as shown in the Democratic Republic of the Congo, Malawi, Mozambique, Niger, and Nigeria in relation to iCCM [71]. Digital data collection also provides opportunities for improvement of routine data collection, quality, and use [72, 73].

Maher and Cometto (2016) [74] have pointed out that research on CHWs should be focused not only on effectiveness (what works) but also on contextual factors and enablers (how, for whom, under what circumstances). George et al. (2018) [75] argue—and we underline this—that efforts to strengthen CHW programmes must “guard against the hubris of relying on a single approach or hierarchy of evidence”. Instead, understanding the implementation realities of CHW programmes will require “humility in understanding communities as social systems, the complexity of the interventions they engage with and the heterogeneity of evidence needs that address the implementation challenges faced”. Most research on CHW programmes has had a narrow focus on a specific disease or intervention, and research on large-scale integrated CHW programmes remains limited. The WHO guideline calls for new health policy and systems methodologies such as implementation research, systems thinking tools, process monitoring, and rapid synthesis of available research [17]. Agarwal et al. (2019) [76] have carried out a literature and consultative review to identify evidence gaps about community health systems and to propose a research agenda to strengthen CHW programmes. Priority research questions they identified included queries on (1) the most effective and efficient supervision and monitoring systems to improve CHW performance, (2) the added value of the use of digital technologies in supervision and monitoring, (3) policies, financing, and governance structures required to support and sustain CHW programmes, (4) CHW models that improve cost-effectiveness and quality of care, and (5) which combinations of training, incentives, and career growth opportunities increase CHW motivation and retention.

Looking at the attributes of individual CHW performance, community-level outcomes and impact at the population level (Tables 1–2, Fig. 1), it is obvious that the academic literature addresses many of these, but others are relatively neglected. More attention to community-level outcomes, such as empowerment and trust, is needed when we aim to fully understand how performance is shaped in different contexts. We also need to recognize that certain CHWs working for a programme may face different factors that influence their performance than other CHWs in the same programme and therefore need different types of support; there is no one-size-fits-all approach to improving CHW performance. As there is evidence that CHW programmes reduce inequities in access to services and promote inclusion of marginalized or vulnerable groups [77], there is also a need to disaggregate metrics to be able to assess for equity. Lastly, as referred to above, it is important to improve methods in assessing cost-effectiveness of CHW programmes [78].

**Table:** 

Key message 6	
CHW programme assessments ideally need to be based on data derived from a mix of reliable sources, obtained using a mix of methods	

## Conclusion

Assessments of CHW programme performance can have various approaches and foci according to the programme and its context. Given the fact that CHW programmes are complex entities and part of health systems, their assessment ideally needs to be based on data derived from a mix of reliable sources. Assessments should be focused not only on effectiveness (what works) but also on contextual factors and enablers (how, for whom, under what circumstances). Serious attention to assessments is instrumental for continually innovating, upgrading, and improving CHW programmes at scale. Programmes will need steadily increasing funding to be able to respond to the gaps and needs identified. Now is the time for a new frontier of implementation research for strengthening CHW programming.

## Data Availability

Data sharing is not applicable to this article as no data sets were generated or analysed.

## Appendix

See Table 4.

**Table 4:** The overview below is derived from the book “Health for the People: National Community Health Worker Programs from Afghanistan to Zimbabwe” (2020). It contains basic information on availability of information systems and national performance assessments of the respective CHW programs. Where cells are left blank, this means No or Unknown

Country	(Selected) household and service delivery data collected by CHWs feed into national level information systems	Large scale national CHW program performance assessment(s) conducted
Afghanistan	Yes	Yes [49]
Bangladesh		
Brazil	Yes	Yes [79]
Ethiopia	Yes	Yes [48]
Ghana	Yes	
India		Yes [80]
Indonesia		
Iran	Yes	
Kenya	Yes	Yes [81]
Liberia	Yes	
Madagascar	Yes	
Malawi	Yes	
Mozambique		
Myanmar		
Nepal	Yes	Yes [82]
Niger		
Nigeria	Yes	
Pakistan	Yes	Yes [83, 84]
Rwanda	Yes	Yes [85]
Sierra Leone	Yes	
South Africa		
Tanzania	Yes	
Thailand	Yes	
Uganda		
Zambia	Yes	
Zimbabwe		

## Abbreviations

AIM: Assessment and Improvement Matrix
ANC: Antenatal care
ASHA: Accredited social health activist
CHC: Community health committee
CHIS: Community health information system
CHW: Community health worker
COVID: Coronavirus disease
DHS: Demographic and health survey
FCHV: Female community health volunteer
iCCM: Integrated community case management
LMICs: Low- and middle-income countries
LQAS: Lot quality assurance sampling
NAP: National evaluation platform
PHC: Primary healthcare
RCT: Randomized controlled trial
TB: Tuberculosis
UNICEF: United Nations Children’s Fund
